# Assessment of the Effects of Structural Modification of *Gastrodia elata* Polysaccharide on Anti-Breast Cancer Activity Using Asymmetrical Flow Field-Flow Fractionation

**DOI:** 10.3390/molecules28124669

**Published:** 2023-06-09

**Authors:** Xiaoying Liu, Yuwei Dou, Tingting Hao, Mu Wang, Liu Yang, Hailiang Zheng, Hongmei Liu, Haiyang Dou

**Affiliations:** 1 Key Laboratory of Pathogenesis Mechanism and Control of Inflammatory-Autoimmune Disease of Hebei Province, School of Basic Medical Sciences, Hebei University, Baoding 071000, China; lxy805912@163.com (X.L.); m18586146062@163.com (Y.D.);; 2 Key Laboratory of Medicinal Chemistry and Molecular Diagnosis of Ministry of Education, College of Chemistry and Materials and Science, Hebei University, Baoding 071002, China; 13206642370@163.com (T.H.); 18931233885@126.com (H.L.); 3 Clinical Laboratory, Affiliated Hospital of Hebei University, Baoding 071000, China

**Keywords:** *Gastrodia elata* polysaccharide, field-flow fractionation, modification, structure-bioactivity relationship

## Abstract

*Gastrodia elata* (“Tian Ma” in Chinese) is used as a food and medical ingredient in traditional Chinese medicine. In this study, to enhance the anti-breast cancer activity of *Gastrodia elata* polysaccharide (GEP), GEPs were modified via sulfidation (SGEP) and acetylation (AcGEP). The physicochemical properties (such as solubility and substitution degree) and structural information (such as molecular weight *M*_w_ and radius of gyration *R*_g_) of GEP derivatives were determined by Fourier transformed infrared (FTIR) spectroscopy and asymmetrical flow field-flow fractionation (AF4) coupled online with multiangle light scattering (MALS) and differential refractive index (dRI) detectors (AF4-MALS-dRI). The effects of the structural modification of GEP on the proliferation, apoptosis, and cell cycle of MCF-7 cell were studied systematically. The ability of MCF-7 cell for the uptake of GEP was studied by laser scanning confocal microscopy (LSCM). The results suggested that the solubility and anti-breast cancer activity of GEP were enhanced and the average *R*_g_ and *M*_w_ of GEP decreased after chemical modification. The AF4-MALS-dRI results showed that the chemical modification process simultaneously caused the degradation and aggregation of GEPs. The LSCM results revealed that more SGEP can enter the MCF-7 cell interior compared with AcGEP. The results indicated that the structure of AcGEP could play a dominating role in antitumor activity. The data obtained in this work can be used as a starting point for investigating the structure-bioactivity of GEPs.

## 1. Introduction

*Gastrodia elata* (“Tian Ma” in Chinese) is used as a food and medical ingredient in traditional Chinese medicine. Recently, *Gastrodia elata* polysaccharides (GEPs), one of the major active components in *Gastrodia elata*, have been demonstrated to exert several pharmacological effects, such as antitumor [1], antioxidant [2], immunomodulatory [3], and neuroprotective activities [4,5]. Several studies have suggested that the biological activities of polysaccharides are closely related to their structure and physicochemical properties, including water solubility, degree of branching, molecular weight (*M*_w_), and monosaccharide composition [6,7,8]. The modification of polysaccharides, including chemical, physical, and biological methods, could change their physicochemical properties, thus enhancing their biological activity [9,10,11]. Generally, physical and biological modifications only change the *M*_w_ of polysaccharides, thereby altering their physicochemical properties and biological activities. Chemical modification is a commonly used method, which can change the structures of polysaccharides by introducing substituent groups [12,13,14]. Several studies have verified that chemical modification could improve the biological activity of polysaccharides [15,16,17]. Chen et al. modified *Ganoderma lucidum* polysaccharides by acetylation and carboxymethylation, and the antioxidant and immunomodulatory activities of the modified products were significantly enhanced [15]. Wang et al. found that the sulfated and carboxymethylated polysaccharides of *Ganoderma lucidum* enhanced their antitumor activity [17]. However, few studies have chemically modified GEP and studied the biological activity of its derivatives. Furthermore, how GEP and its derivatives exert their antitumor activities inside and/or on the surface of cells is unclear, which is partly due to their complex structure. Therefore, the structural characterization of GEP and its derivatives is necessary.

It is well known that polysaccharides cannot be monitored by fluorescence and ultraviolet (UV) detectors because of the absence of fluorescent and UV absorption groups. The *M*_w_ distribution of polysaccharides is frequently analyzed by size exclusion chromatography (SEC) coupled with multiangle light scattering (MALS) and differential refractive index (dRI) detectors. For ultrahigh *M*_w_ or branched polymers, this method may not be sufficient, because the column packing materials of SEC would lead to the shear degradation of polysaccharides and the irreversible interaction between the column packing materials and the polysaccharides, and then falsify the determined *M*_w_ distribution. Asymmetrical flow field-flow fractionation (AF4) is a gentle separation technique and offers the possibility to overcome the problems of SEC due to the absence of a stationary phase or packing materials in AF4 [18]. In our previous work [19], AF4-MALS-dRI was proven to be a promising tool for understanding the relationships between the structure and anti-breast cancer activity of GEPs. The objectives of the current study were to enhance the anti-breast cancer activity of GEPs by chemical modification and to further explore the relationships between the structure and anti-breast cancer activity of GEPs’ derivatives.

## 2. Results and Discussion

### 2.1. Modification of GEP

It has been reported that the antitumor activity of polysaccharides can be enhanced by structural modification [20]. In this study, a sulfated derivative (SGEP) and an acetylated derivative (AcGEP) of GEP were prepared. The total polysaccharide content of GEP was determined using the phenol–sulfuric acid method to be 96.17 ± 3.82%. The degree of substitution (DS) and solubility of GEP and its derivatives were determined and listed in Table 1. The DS of SGEP and AcGEP were 0.37 ± 1.39% and 0.22 ± 0.82%, respectively. The solubility of GEP was improved after sulfation and acetylation modification. The solubility of GEP increased from 15.3 mg/mL to 35.7 mg/mL when acetyl group was introduced into the GEP molecule. This is because the introduction of sulfate or acetyl groups can weaken the intermolecular and intramolecular interactions, which contributes to the exposure of more hydrophilic groups of polysaccharides, thereby increasing the solubility of GEP in water [9].

**Figure 1:**
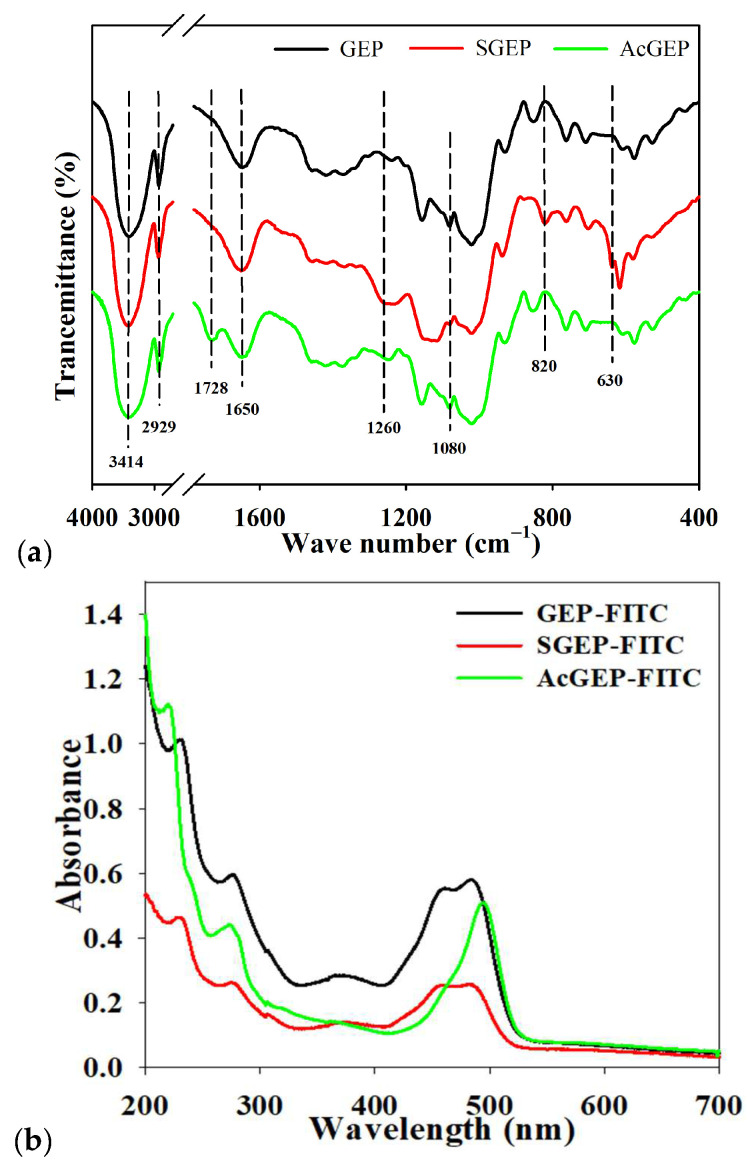
FTIR spectra of GEP, SGEP, and AcGEP (**a**); UV-Vis spectra of GEP-FITC, SGEP-FITC, and AcGEP-FITC (**b**).

FTIR is commonly used to characterize the functional groups of polysaccharides. The FTIR spectra of GEP, SGEP, and AcGEP are illustrated in Figure 1a. The characteristic absorption bands of polysaccharide at 3414, 2929, and 1080 cm^−1^ were clear for the three samples. These bands are attributed to O–H bending vibration, C–H stretching vibration, and C–O stretching vibration, respectively [21]. Polysaccharides can be easily hydrated due to their affinity for water and this is consistent with the presence of the water absorption band at 1650 cm^−1^. The intensity of band at 1650 cm^−1^ for SGEP was the strongest among the three samples. Two new absorption bands at 1260 and 820 cm^−1^ appeared in the FTIR spectrum of SGEP, which are attributed to the characteristic absorption bands of an asymmetrical S=O stretching vibration and a symmetrical C–O–S vibration associated with a C–O–SO_3_ group, respectively [22]. For AcGEP, a new band at 1728 cm^−1^ was observed, which is assigned to the C=O stretching vibration [23]. The FTIR results demonstrated that the sulfate and acetyl groups were successfully introduced into GEP molecules.

### 2.2. AF4 Analysis of GEP and Its Derivatives

**Figure 2:**
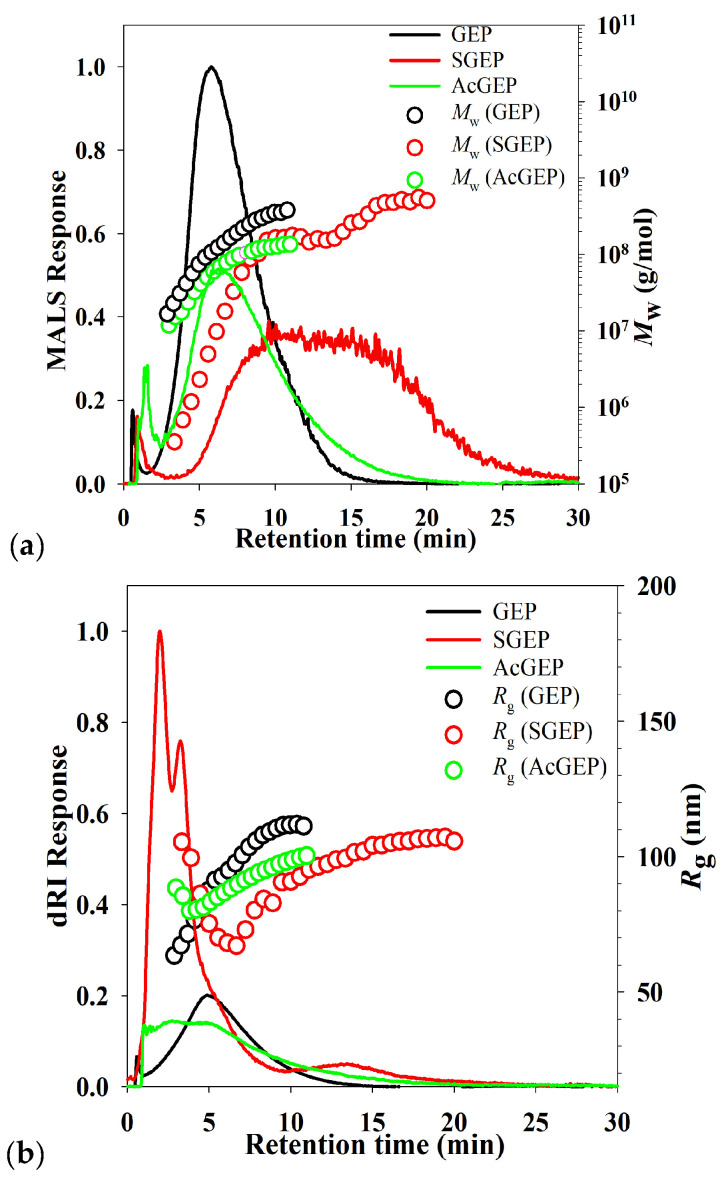

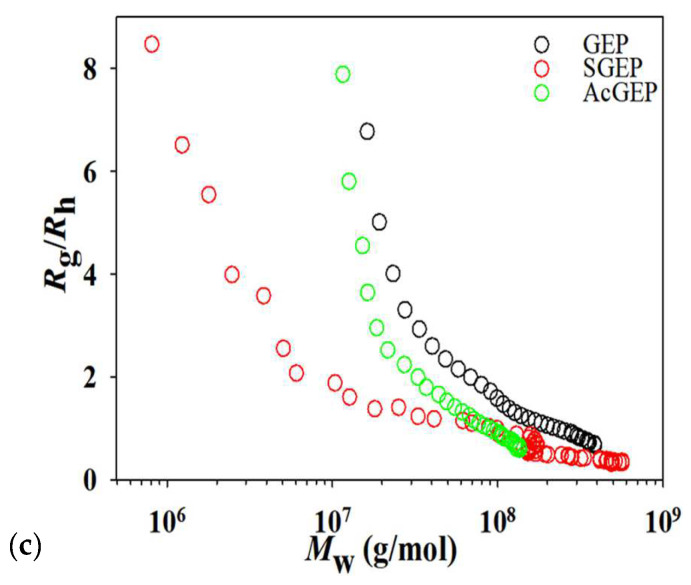
AF4-MALS-dRI fractograms, *M*_w_ distributions (**a**), *R*_g_ distributions (**b**), and *R*_g_/*R*_h_ (**c**) of GEP and its derivatives.

AF4-MALS-dRI was employed for the characterization of the *M*_w_, the radius of gyration (*R*_g_), and molecular conformation of GEP and its derivatives. It can be seen in Figure 2a that the *M*_w_ of the three samples gradually increased with increasing retention time as expected by AF4 normal elution mode [24]. After modification, the intensities of the dRI signal of GEP’s derivatives at low retention time (less than 4 min in Figure 2b) increased, which indicates a degradation in GEP during modification process. Meanwhile, a new population with a large size peaking at approximately 14 min (Figure 2b) was observed for SGEP, indicating a formation of aggregates. The result suggested that the degradation and aggregation of GEP were a kinetic equilibrium process during SGEP preparation [25]. The average *R*_g_ and *M*_w_ of the three samples are listed in Table 1. SGEP and AcGEP had the smallest average *R*_g_ (85.8 nm) and the smallest *M*_w_ (6.4 × 10^7^ g/mol), respectively. The ratio of *R*_g_ to *R*_h_ can be used to evaluate the conformation of molecules [25]. The value of *R*_g_/*R*_h_ less than 0.7 corresponds to highly expanded macromolecules. The value of *R*_g_/*R*_h_ (1.0–1.5) corresponds to branched molecules. The value of *R*_g_/*R*_h_ (1.5–2.1) corresponds to random coil conformation. The value of *R*_g_/*R*_h_ larger than 2.0 corresponds to rod-like conformation. Figure 2c shows that the trend of *R*_g_/*R*_h_ of the three samples was similar over entire *M*_w_ distribution. For SGEP, the population with *M*_w_ less than 6.0 × 10^7^ g/mol had more elongated conformation. This may be due to the electrostatic repulsion and steric hindrance of the sulfate groups on the polysaccharide chain, which makes the population with small *M*_w_ present a relatively extended chain conformation [26].

### 2.3. Anti-Breast Cancer Activity of GEP and Its Derivatives

**Figure 3:**
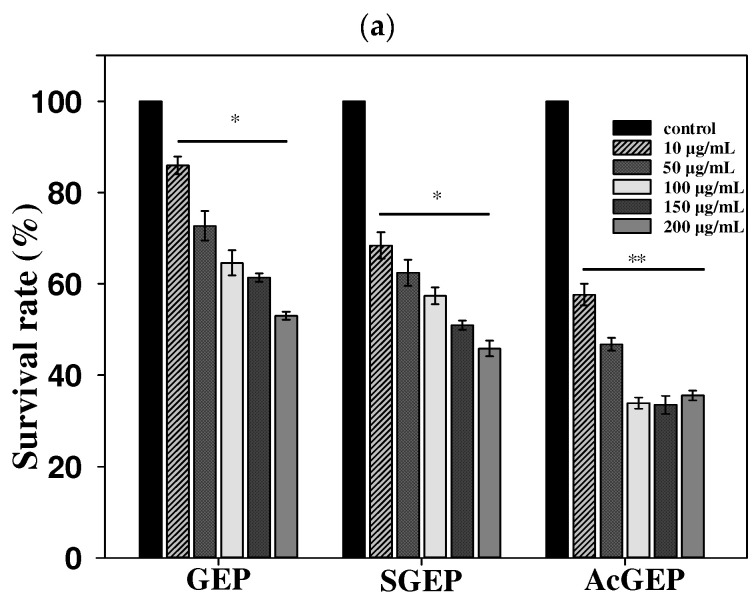

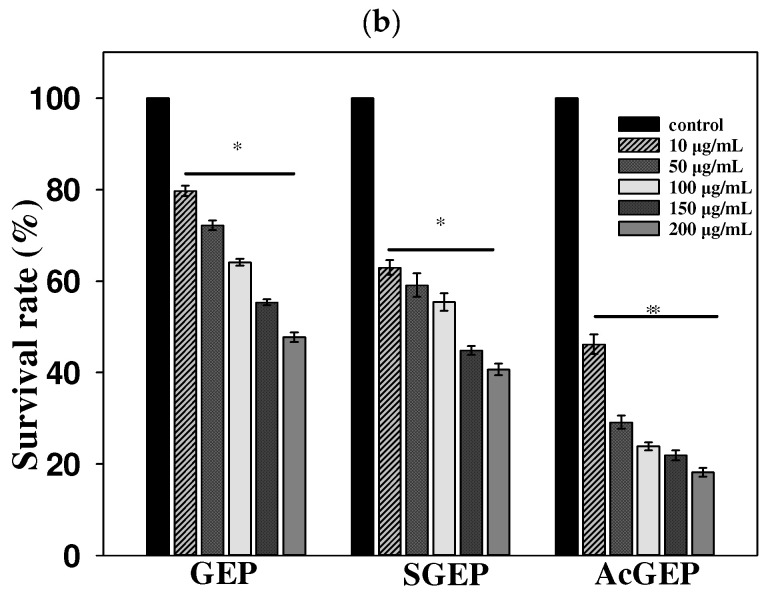
Effect of GEP and its derivatives on MCF-7 cell viability detected by MTT assay for 24 h of drug action (**a**) and 48 h of drug action (**b**). * *p* < 0.05 and ** *p* < 0.01 compared with control group.

MTT assay is often used to assess cell viability, activation, and proliferation. The MCF-7 cell proliferation rate was utilized to assess the anti-breast cancer activity of GEP and its derivatives. Figure 3 shows the effect of GEP and its derivatives with different concentrations on MCF-7 cell viability detected by MTT assay. With increasing concentration and incubation time of GEP and its derivatives, the survival rate of MCF-7 cells decreased, demonstrating that GEP and its derivatives have an inhibition effect on MCF-7 cells in dose- and time-dependent manners. Moreover, the results indicated that the anti-breast cancer activity of GEP was enhanced after structural modification (i.e., sulfation and acetylation), especially for acetylated GEP. It is reported that the introduction of sulfate and acetyl groups into polysaccharides can improve their solubility, which is beneficial to their bioactivities [27,28]. The solubility of GEP was determined to be 15.3 mg/mL (Table 1). GEP and its derivatives were completely dissolved in the concentration investigated in this study. Thus, the solubility of GEP cannot be used to explain the enhancement in anti-breast cancer activity. One possible explanation is that the change in GEP structure during modification may contribute to the enhancement of this molecule’s anti-breast cancer activity. AcGEP had the smallest *M*_w_ and the best anti-breast cancer activity among the three GEP samples. Although this conclusion is only proposition, the knowledge gained from this study could help to elucidate the roles of the structure of GEP in its bioactivity.

**Figure 4:**
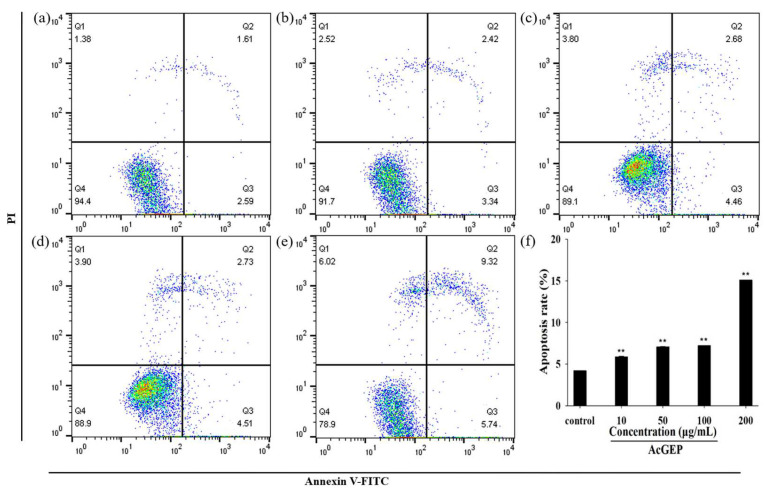
FACS results of cell apoptosis in MCF-7 cells treated with AcGEP for 48 h (**a**–**e**) and column bar graph of apoptotic cells (**f**). Representative dot plots of Annexin V-FITC/PI-stained cells detected by FACS after treatment with different concentrations of AcGEP (0, 10, 50, 100, 200 μg/mL) for 48 h. The untreated cells were used as control group. Data were expressed as the mean ± SD. ** *p* < 0.01 compared with the control group.

**Figure 5:**
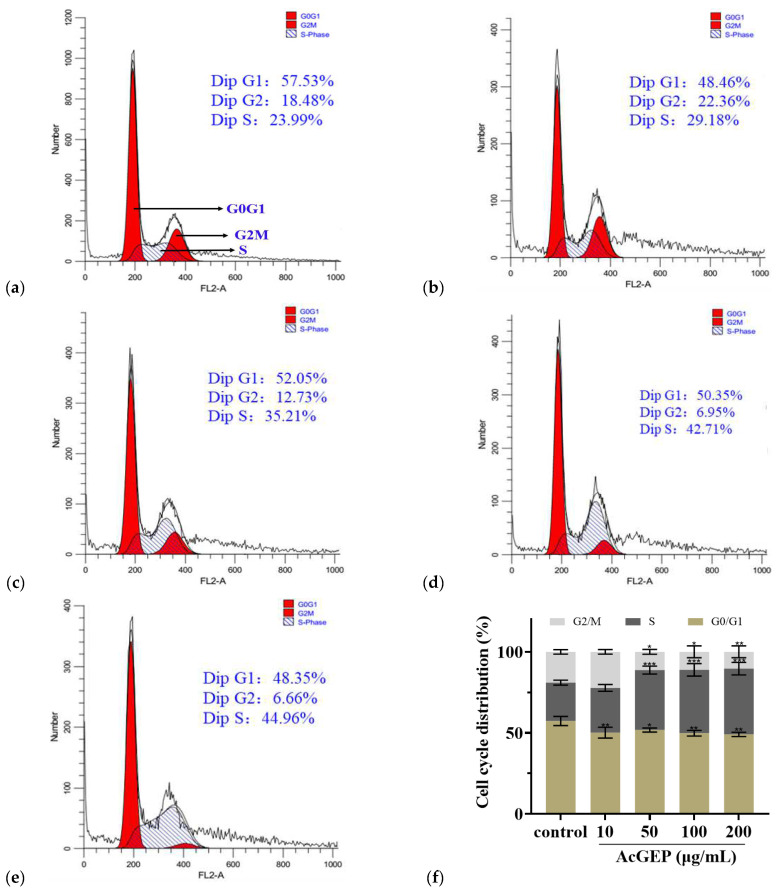
Effect of AcGEP on MCF-7 cell cycle. Control cells (**a**). Cells treated with 10 μg/mL AcGEP (**b**). Cells treated with 50 μg/mL AcGEP (**c**). Cells treated with 100 μg/mL AcGEP (**d**). Cells treated with 200 μg/mL AcGEP (**e**). Quantitative presentation of cycle induced by AcGEP in MCF-7 cells (**f**). * *p* < 0.05, ** *p* < 0.01 and *** *p* < 0.01 compared with control group.

Cell apoptosis plays an indispensable role in tumor progression. In this study, the apoptosis of MCF-7 cells treated with different concentrations of AcGEP was analyzed by Annexin V-FITC/PI kit according to the manufacturer’s specification. It can be seen from Figure 4a–e that AcGEP induced MCF-7 cells apoptosis in a dose-dependent manner. A higher concentration of AcGEP induced more apoptotic cells (Q2 and Q3 quadrants) and necrotic cells (Q1 quadrants). The apoptotic rate of MCF-7 cells was calculated based on late apoptotic cells and early apoptotic cells. Figure 4f shows that there are quite significant differences (*p* < 0.01) in the percentage of apoptotic MCF-7 cells in the concentration range of AcGEP investigated in this study, compared with the control group. Figure 5 shows the effect of AcGEP on the MCF-7 cell cycle. The results suggested that the MCF-7 cell cycle was arrested at the S phase after AcGEP treatment. In our previous study, it was found that GEP can arrest MCF-7 cell cycle at the G2/M phase [19]. The results indicated that the structure of GEP plays an important role in MCF-7 cell cycle.

**Figure 6:**
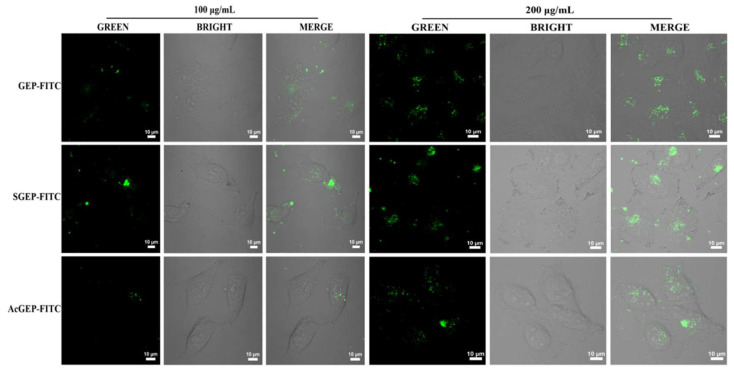
Fluorescence images of MCF-7 cells treated with 100 μg/mL and 200 μg/mL of GEP-FITC, SGEP-FITC, and AcGEP-FITC.

The fluorescent-labeled polysaccharides can be directly observed by fluorescence laser scanning confocal imaging, which is conducive to the exploration of the pharmacokinetic mechanism of polysaccharides in vitro and in vivo. After introducing a secondary amino group into the polysaccharide chain, GEP can directly react with FITC. The orange-yellow powder of GEP-FITC, SGEP-FITC, and AcGEP-FITC were obtained. Figure 1b shows that the fluorescent-labeled GEP samples had a characteristic absorption peak of FITC at 490 nm [29], indicating that the fluorescein (FITC) was successfully linked with GEP samples. Figure 6 shows the fluorescence images of MCF-7 cells treated by GEP and its derivatives labeled with FITC. The fluorescence signal was observed for the three labeled GEP samples in the green mode and MCF-7 cells can be clearly observed in the bright mode. After merging both green and bright images, the fluorescence signal in the MCF-7 cells was observed in the merge mode. The intensity of fluorescence signal in the MCF-7 cells enhanced with increasing concentration of GEP samples (200 μg/mL). The results suggested that GEP can enter the MCF-7 cell interior.

## 3. Materials and Methods

### 3.1. Materials

*Gastrodia elata* (dried rhizomes) were obtained from Zhaotong Laomao Food Co., Ltd. (Zhaotong, China). Deionized water was obtained from a UPR-Ⅱ-10T Ultra-Pure Water system (Ulupure Co., Ltd., Chengdu, China). Acetic anhydride and barium chloride were purchased from Tianjin Damao Chemical Reagent Factory (Tianjin, China). Ammonium sulfate and potassium sulfate were purchased from Tianjin Kaitong Chemical Reagent Co., Ltd. (Tianjin, China). Sodium bicarbonate was obtained from Tianjin Fuchen Chemical Reagent Co., Ltd. (Tianjin, China). Ethanol was obtained from Tianjin Kermel Chemical Reagent Co., Ltd. (Tianjin, China). Sulfuric acid (H_2_SO_4_) was obtained from Beijing Chemical Co., Ltd. (Beijing, China). Potassium bromide (KBr) was procured from Tianjin Nuoleixinda Technology Co., Ltd. (Tianjin, China). *N*-butyl alcohol, trichloroacetic acid, disodium phosphate, sodium dihydrogen phosphate, tyramine, sodium cyanoborohydride (NaBH_3_CN), sodium hydroxide (NaOH), hydrochloric acid (HCl), and gelatin were obtained from Shanghai Macklin Biochemical Co., Ltd. (Shanghai, China). Dulbecco’s modified Eagle’s medium (DMEM) was purchased from Gibco Life Technologies (Grand Island, NY, USA). Fetal bovine serum (FBS) was procured from Biological Industries (Kibbutz Beit-Haemek, Israel). Phosphate buffer solution, 0.25% trypsin, 3-(4,5-dimethyl-2-yl)-2,5-diphenyltetrazolium bromide (MTT), and dimethyl sulfoxide (DMSO) were procured from Beijing Solarbio Science and Technology Co., Ltd. (Beijing, China). Annexin V-FITC/PI (propidium iodide) apoptosis detection kit was obtained from Vazyme Biotech Co., Ltd. (Nanjing, China). Cell cycle detection kit was procured from Beyotime Biotechnology Co., Ltd. (Shanghai, China).

### 3.2. Extraction of Polysaccharide from Gastrodia elata

The extraction of GEP was performed with a previously reported method [19]. Briefly, the dried *Gastrodia elata* (50 g) was powdered and defatted twice with 80% (*v*/*v*) ethanol for 2 h each time. Skimmed *Gastrodia elata* powder (10 mg) was added into deionized water (300 mL) (1:30 *w*/*v*) in a 500 mL beaker, followed by sonicating in an ultrasound bath (Kunshan Ultrasound Instrument Co., Ltd., Kunshan, China) at 70 °C at a power of 200 W and frequency of 40 kHz for 30 min. The ultrasound extraction process was repeated three times. The extract was centrifuged by an X-15R centrifuge (Beckman Coulter, Inc., Palo Alto, CA, USA). The supernatant was concentrated by a RE-2000A rotary vacuum evaporator (Yarong Biochemical Instrument Factory, Shanghai, China). Then, 95% (*v*/*v*) ethanol was added into the concentrated solution and placed at 4 °C overnight for polysaccharide precipitation. The precipitate of GEP was centrifuged and freeze-dried by a MODULYOD freeze-dryer (Thermo Fisher Scientific, Waltham, MA, USA). In this study, the phenol–sulfuric acid method [30] was employed to determine total polysaccharide content of GEP. 

### 3.3. Preparation of Sulfated Gastrodia elata Polysaccharide

The sulfated GEP (SGEP) was prepared according to a previous method with some modifications [31]. Briefly, 2.5 mL of *n*-butanol alcohol and 7.5 mL of concentrated H_2_SO_4_ were slowly added into the stoppered conical flask placed in an ice-water bath in advance. Then, 125 mg of ammonium sulfate was added and stirred for 10 min in an ice-water bath. After that, 500 mg of GEP was slowly added and allowed to react for 3 h in an ice-water bath. Then, the pH of the solution was adjusted to 7.0 by using NaOH (5.0 mol/L) and centrifuged at 3000 rpm for 5 min. Finally, the supernatant was freeze-dried to obtain SGEP.

The degree of substitution (DS) of SGEP was determined by the barium chloride-gel method with slight modifications [32]. Briefly, potassium sulfate (54.4 mg) was dissolved in 50.0 mL of HCl (1.0 mol/L) to obtain a standard solution. Standard solution with different volumes (0.0 mL, 0.2 mL, 0.4 mL, 0.8 mL, and 1.0 mL) and 1.0 mol/L HCl were added to a test tube to obtain 1.0 mL of the resulting volume of the solution. Barium chloride–gelatin solution (0.5 mL, 0.5%, *w*/*v*) and trichloroacetic acid (3.5 mL, 3.0%, *w*/*v*) were added and shaken thoroughly. The resulting solution was left to rest at room temperature for 15 min. The absorbances of the solution (*A*_1_) and the blank control (*A*_2_) were measured at a wavelength of 360 nm. The blank control was the same as the above procedure except that barium chloride was replaced by the gelatin solution. A standard curve of the absorbance *A*_0_ = (*A*_1_ − *A*_2_) and concentration of ${\mathrm{SO}}_{4}^{2-}$ was established. Sulfate content of SGEP was determined according to the method described above when the standard solution was replaced by SGEP solution. The DS of SGEP was calculated by Equation (1):

(1) $$\mathrm{DS}=1.62\times \frac{S}{32-1.02\times S}\;$$

where *S* is the content of sulfur atom converted from the content of sulfate in the sample.

### 3.4. Preparation of Acetylated Gastrodia elata Polysaccharide 

The acetylated GEP (AcGEP) was prepared according to a previous method with slight modifications [33]. Briefly, 500 mg of GEP was dissolved in 10.0 mL of deionized water. The pH of the solution was adjusted by 2.5 mol/L NaOH and stirred at 30 °C for 10 min. Then, 4.0 mL of acetic anhydride was added to the solution and reacted in a water bath at 30 °C for 3 h. The pH of the reaction solution was controlled in the range of 8.0–8.5 by adding 2.5 mol/L NaOH occasionally. After that, the reaction solution was neutralized with 1.0 mol/L HCl solution. Subsequently, the reaction solution was freeze-dried to obtain AcGEP.

The DS of AcGEP was determined by the acid-base titration method [34]. Briefly, 10 mg of AcGEP was dissolved in 10.0 mL NaOH (0.01 mol/L). The AcGEP solution with several drops of phenolphthalein was titrated with HCl (0.01 mol/L). Note that the polysaccharide could consume the alkaline solution. Thus, GEP solution was used as a blank control for the titration process. The DS of AcGEP was calculated according to Equations (2) and (3):

(2) $$A\%=\left(C_{0}V_{0}-C_{1}V_{1}\times 0.043\times \frac{100}{m}\right)$$

(3) $$\mathrm{DS}=\frac{132\;A}{4300-42\;A}$$

where *A* is the content of acetyl in the sample, *C*_0_ and *C*_1_ are the concentrations of NaOH and HCl, respectively. *V*_0_ and *V*_1_ are the consumed volumes of NaOH and HCl, respectively. Additionally, *m* is the sample mass.

### 3.5. Determination of Solubility of GEP and Its Derivatives

A previously reported method was used to determine the solubility of GEP and its derivatives [35]. Briefly, 200 mg of polysaccharide sample was added in 5.0 mL deionized water and stirred at room temperature for 24 h. Then, the mixture was centrifuged and the supernatant was removed. The residue was freeze-dried and accurately weighed. The solubility of the three samples was calculated by the following Equation (4):

(4) $$\mathrm{Solubility}\;\left(\frac{\mathrm{mg}}{\mathrm{mL}}\right)=\frac{m_{0}-m_{1}}{V}$$

where *m*_0_ is the initial mass of GEP and its derivatives (i.e., 200 mg), *m*_1_ is the remaining mass after centrifugation. *V* is the volume of deionized water (i.e., 5.0 mL).

### 3.6. Fourier Transformed Infrared (FTIR) Spectroscopy

The FTIR spectra of GEP and its derivatives were acquired using a TENSOR II spectrometer (Bruker Corporation, Ettlingen, Germany) in the wavenumber range of 400–4000 cm^−1^. The sample was mixed with KBr to a ratio of approximately 1:100 (*w*/*w*). The mixture was ground to a homogenous powder and then pressed into tablets. The FTIR spectra of the samples were recorded with a resolution of 4 cm^−1^ and 32 scans.

### 3.7. AF4 Analysis of GEP and Its Derivatives

In the present study, the AF4 analysis of GEP and its derivatives were carried out by an Eclipse AF4 system (Wyatt Technology, Dernbach, Germany). AF4 was connected to a DAWN EOS MALS detector (Wyatt Technology, Santa Barbara, CA, USA) and a RID-20 dRI detector (Shimadzu, Kyoto, Japan). In the AF4 analysis of samples, the carrier liquid was ultrapure water containing 5.0 mM NaNO_3_ (pH 7.0). The carrier liquid was delivered into the AF4 channel by an Agilent 1260 Infinity Ⅱ pump (Agilent Technologies, Waldbronn, Germany). The Eclipse AF4 long channel (LC, 265 mm length) was assembled with a 350 μm thickness mylar spacer and a regenerated ultrafiltration cellulose membrane with a molecular weight cutoff of 10 kDa. The concentration of SGEP solution was 1.0 mg/mL, while the concentration of GEP and AcGEP solutions was 0.5 mg/mL. The sample (50 μL) was injected into the channel at a flow rate of 0.2 mL/min for 2.0 min. The detector flow rate was kept constant at 1.0 mL/min. The cross-flow rate for the analysis of SGEP started at 1.5 mL/min and exponentially decreased to 0.05 mL/min with a half-life of 1.0 min, while the cross-flow rate for the analysis of GEP and AcGEP started at 0.2 mL/min and exponentially decreased to 0.05 mL/min with a half-life of 2.0 min. AF4-MALS-dRI data were analyzed by the Astra 6.1.7 software (Wyatt Technology, Santa Barbara, CA, USA) with Debye mode and a fit degree of 2. 

### 3.8. Anti-Breast Cancer Activity of GEP and Its Derivatives

#### 3.8.1. Cell Culture and MTT Assay

Human breast cancer MCF-7 cell line was procured from the National Experimental Cell Resource Sharing Platform. MCF-7 cells were cultured in DMEM supplemented with FBS at 37 °C in a 5% CO_2_ incubator (Thermo Fisher Scientific, Marietta, OH, USA) until 70–90% confluency was achieved. 

The antitumor activities of GEP and its derivatives were determined using the MTT assay under the same conditions. Briefly, MCF-7 cells were seeded in a 96-well plate at a density of 5 × 10^3^ cells per well in 100 μL DMEM and cultured at 37 °C in a 5% CO_2_ incubator when the cells reached the logarithmic growth stage. When the confluency of MCF-7 cells reached approximately 70%, the cells were divided into the negative control group and the experimental group (5 duplicate wells per group). The cells of the control and experimental groups were treated with GEP and its derivative DMEM solutions for 24 and 48 h, respectively. The resulting concentrations of GEP and its derivative were 0, 10, 50, 100, 150, and 200 μg/mL, respectively. Then, 20 μL of MTT (5.0 mg/mL) was added to each well for another 4 h. After that the supernatant (i.e., 100 μL DMEM and 20 μL MTT) was removed and 200 μL of DMSO was added to dissolve formazan. Finally, the plate was shaken for 15 min in the dark and the absorbance of each well was measured at a wavelength of 490 nm by a microplate reader. The proliferation of MCF-7 cells treated with GEP and its derivatives was calculated according to Equation (5):

(5) $$\mathrm{Cell}\;\mathrm{survival}\;\mathrm{rate}=\frac{\mathrm{OD}\;\mathrm{sample}}{\mathrm{OD}\;\mathrm{control}}\times 100\%\;$$

where OD_sample_ and OD_control_ are the optical density value of the experimental group and control group, respectively.

#### 3.8.2. Cell Apoptosis Assay

The cell apoptosis rates were determined using Annexin V-FITC/PI Apoptosis kit by fluorescence-activated cell sorting (FACS) (FACS-Calibur™, BD Bio-Sciences, San Jose, CA, USA). The MCF-7 cells incubated in advance were digested with trypsin, washed with PBS, and centrifuged (1000 rpm for 5 min). Then, the cells were collected and resuspended with 100 μL binding buffer and transferred to a tinfoil-wrapped flow tube, followed by Annexin V-FITC and PI staining for 10 min at 37 °C in the dark. Finally, the cell apoptosis rates were determined by FACS within 1 h.

#### 3.8.3. Cell Cycle Analysis

Cell cycle was investigated by PI staining assay kit, including ribonuclease A. After incubation, the control and treated cells were collected, resuspended, and centrifuged. Then, the supernatant was aspirated and discarded. After that, the cells were fixed with prechilled 70% (*v*/*v*) ethanol at 4 °C for 12 h. Then, the fixed cells were centrifuged at 1000 rpm for 5 min and resuspended with prechilled PBS, centrifuged, and discarded the supernatant. Finally, the cells were stained with the staining solution of PI prepared in advance according to the manufacturer’s instructions and incubated at 37 °C for 30 min in the dark. The percentages of stained cells in each phase were measured with FACS and analyzed using Flowjo software (Becton Dickinson and Company, San Jose, CA, USA).

#### 3.8.4. Uptake of Fluorescent-Labeled GEP and Its Derivative by MCF-7 Cells

The fluorescent-labeled polysaccharides were prepared according to a previously described method with slight modifications [36]. Briefly, GEP or its derivative powders (400 mg) were dissolved with 15 mL of phosphate buffer (0.2 mol/L, pH 8.5) in a test tube, and then tyramine (400 mg) was added to each polysaccharide solution and reacted at room temperature for 12 h. After that, NaBH_3_CN (150 mg) was added to the test tube and reacted at 37 °C for 96 h with occasional shaking. Under the catalysis of NaBH_3_CN, the reducing terminal aldehyde group of GEP and its derivative reacted with tyramine. After centrifugation, the supernatant was freeze-dried to obtain the product, and then the product was dissolved into 10.0 mL deionized water (pH 8.0–10.0, adjusted by 0.5 mol/L NaHCO_3_), followed by the addition of 30 mg FITC to the solution. The secondary amino group of the product and the cyano-group of FITC underwent the nucleophilic reaction to generate the fluorescent-labeled GEP, SGEP, and AcGEP (denoted as GEP-FITC, SGEP-FITC, and AcGEP-FITC, respectively). The mixture was incubated at room temperature for 24 h in the dark. After reaction, 95% (*v*/*v*) ethanol was added and left standing for 24 h to obtain GEP-FITC, SGEP-FITC, and AcGEP-FITC. The precipitate was obtained by centrifugation. The precipitate was washed with 95% (*v*/*v*) ethanol until the solution had no fluorescence and then freeze-dried. Finally, fluorescent-labeled polysaccharides were obtained. The fluorescent-labeled products were verified by UV-visible spectrophotometer UV-2550 (Shimadzu, Kyoto, Japan) at 200–900 nm.

To investigate the uptake of GEP by MCF-7 cell, 1.0 mL of GEP-FITC, SGEP-FITC, and AcGEP-FITC solutions with concentrations of 100 μg/mL and 200 μg/mL were added to the experimental group, respectively. The cells were cultured at 37 °C in a 5% CO_2_ incubator for 24 h. Then, the fluorescence imaging was captured using a Zeiss LSM880 + Airyscan laser scanning confocal microscope (excitation at 488 nm and emission collected at 500–625 nm) for fluorescent-labeled GEP samples.

### 3.9. Data Processing

The data were analyzed by SPSS 22.0 software. The data were described as mean ± standard deviation (SD). In this study, one-way ANOVA was used for comparison among multiple groups. LSD-*t* test was used for comparison between two groups. The values of *p* < 0.05 and *p* < 0.01 were considered as statistically significant difference and quite significant difference, respectively. All experiments were performed in triplicate. 

Hydrodynamic radius (*R*_h_) of sample was calculated according to Equation (6) [37]:

(6) $$R_{h}=\frac{kTV^{0}}{\pi \eta V_{c}w^{2}t^{0}}t_{r}$$

where *k* is the Boltzmann constant, *T* is the absolute temperature, *V*^0^ is the channel void volume, *η* is the viscosity of the carrier liquid, *V_c_* is the cross-flow rate, *w* is the channel thickness, and *t*^0^ and *t_r_* are the void time and the retention time, respectively.

## 4. Conclusions

In this study, the sulfated and acetylated GEPs were successfully prepared, as verified by FTIR results. The substitution degrees of SGEP and AcGEP were 0.37 ± 1.39% and 0.22 ± 0.82%, respectively. The solubility of GEP was improved by sulfation and acetylation modification. The AF4-MALS-dRI results showed that the chemical modification process simultaneously causes the degradation and aggregation of GEPs. Overall, the average *R*_g_ and *M*_w_ of GEP decreased after chemical modification. SGEP had the smallest average *R*_g_ (85.8 nm) and a more elongated conformation. AcGEP had the smallest average *M*_w_ (6.4 × 10^7^ g/mol). The MTT results showed that GEP derivatives had better anti-breast cancer activity compared with GEP, especially for AcGEP. Furthermore, the results revealed that AcGEP can induce apoptosis of MCF-7 cells and inhibit proliferation of MCF-7 cells by blocking in the S phase. The confocal fluorescence imaging results revealed that GEP can enter the MCF-7 cell interior. The results indicated that a desired structure of AcGEP (such as *M*_w_ distribution) would be beneficial to its anti-breast cancer activity. However, more in-depth studies are required to understand how the structure of AcGEP affects its bioactivity.

## Figures and Tables

**Table 1:** Characteristics of GEP and its derivatives.

Sample	Substitution Degree	Solubility (mg/mL)	Average *R*_g_ (nm)	Average *M*_w_ (×10^7^ g/mol)
GEP	--	15.3 ± 1.8%	92.4 ± 3.9%	12.9 ± 4.4%
SGEP	0.37 ± 1.39%	35.7 ± 0.6%	85.8 ± 6.0%	9.3 ± 5.5%
AcGEP	0.22 ± 0.82%	24.5 ± 1.0%	88.4 ± 2.6%	6.4 ± 2.8%

## Data Availability

Data will be made available on request.
